# The mobile emergency recovery intervention trial (MERIT): Protocol for a 3-year mixed methods observational study of mobile recovery outreach teams in Nevada’s emergency departments

**DOI:** 10.1371/journal.pone.0258795

**Published:** 2021-10-28

**Authors:** Krysti P. Smith, Roy F. Oman, Minggen Lu, Ashley D. Dawkins, Robert W. Harding, Katherine Hepworth, Karla D. Wagner

**Affiliations:** School of Community Health Sciences, University of Nevada, Reno, NV, United States of America; Monash University Malaysia, MALAYSIA

## Abstract

**Background:**

The Substance Abuse and Mental Health Administration awarded State Targeted Response grants to support states’ efforts to address the opioid epidemic. In Nevada, one component of this grant was mobile recovery outreach teams (MROTs) that utilized peer recovery support specialists to provide care for qualifying patients in emergency departments (EDs). The Mobile Emergency Recovery Intervention Trial (MERIT) is a mixed methods study to assess the feasibility/acceptability and effectiveness of the MROT intervention. This protocol mainly describes the R33 research activities and outcomes. The full protocol can be found protocols.io.

**Methods:**

Data will be derived from state-level data sets containing de-identified emergency department visits, substance use disorder treatment records, and mortality files; in-person mixed methods interviews; participant observation; and self-report process evaluation forms. Primary outcomes include Medication Assisted Treatment (MAT) initiation and non-fatal overdose; secondary outcomes include MAT retention and fatal overdose. Quantitative hypotheses will be tested using generalized linear mixed effects models, Bayesian hierarchical models, and marginal Cox models. Qualitative interview data will be analyzed using an inductive thematic analysis procedure.

**Discussion:**

- It is impossible to conduct a randomized controlled trial of the effectiveness of the MROTs, given the ethical and logistical considerations of this intervention.
- This study’s innovative design employs a mixed methods formative phase to examine feasibility and acceptability, and a quasi-experimental outcomes evaluation phase employing advanced statistical methods to mitigate bias and suggest causal inference regarding the effectiveness of the MROTs.
- Innovative interventions have been deployed in many states; evidence regarding their effectiveness is lacking, but critical to informing an effective public health response to the opioid epidemic.

## Introduction

In 2018, approximately 46,802 reported drug poisoning deaths involved an opioid; as the opioid overdose epidemic continues to be a prevalent public health crisis in the United States, emergency departments throughout the country act as the front-line response for many opioid overdoses [1]. Previous research involving people who inject drugs has shown that after a non-fatal overdose, talking with a spouse or partner, crisis counselor, or hospital staff about substance use disorder treatment was associated with an increased odds of seeking treatment in the 30 days following an overdose [2]. Therefore, ED visits as a result of overdoses can represent an opportunity to provide linkage to care and prevent future overdoses from occurring [3].

In FY2017, the Substance Abuse and Mental Health Administration (SAMHSA) granted almost $500 million dollars to states in the form of State Targeted Response (STR) to the Opioid Crisis grants (TI-17-014), designed to support states’ efforts to address the opioid overdose crisis by increasing access to treatment, targeting unmet treatment needs, and reducing the number of opioid related deaths through increased prevention efforts. The infusion of funds to rapidly expand services created an opportunity for innovative intervention approaches, as well as an opportunity to conduct research evaluating the impact of those approaches on reducing opioid overdose morbidity and mortality.

In Nevada, one high-profile component of the STR was the development of mobile recovery outreach teams (MROTs). MROTs, composed of substance use clinicians and peer recovery support specialists with lived experience of substance use, were developed to provide support for overdose patients and individuals presenting in Nevada’s EDs with primary or secondary diagnoses of opioid use disorder. During the ED interaction, the MROTs provide peer support for patients and facilitate overdose prevention education, naloxone distribution, linkage to care including medication assisted treatment (MAT), and recovery options via a hub and spoke treatment infrastructure supported by the broader Nevada STR program. Following the initial STR activities, additional funds were awarded to the Nevada Department of Health and Human Services, Division of Public and Behavioral Health, Behavioral Health Prevention and Treatment Program by SAMSHA under the Nevada State Opioid Response (SOR) Grant (Grant Number 3 H79 TI081732-01S1). The overarching goal of the SOR grant was to expand the work accomplished by the STR-funded projects and to increase access to overdose prevention, treatment and recovery support services. These funds were used to establish two additional MROTs connected to community agencies outside of the STR efforts.

While there is some evidence from descriptive studies that using a peer-driven intervention model in EDs can be effective, few studies have rigorously evaluated the impact of peer-driven ED interventions on patient outcomes, including linkage to treatment, subsequent overdose, and overdose mortality. Furthermore, integration of new interventions into the complex organizational dynamics of hospitals and EDs can be challenging, requiring formative feasibility and acceptability research to ensure the success of the broader implementation effort, and an evaluation of outcomes to test the impacts of these teams for people who use drugs.

The Mobile Emergency Recovery Intervention Trial (MERIT) is a mixed methods study designed to generate evidence regarding the feasibility/acceptability and effectiveness of the MROT intervention. The initial study was designed in two stages, a 1-year R21 phase to evaluate feasibility/acceptability and a 3-year R33 phase to evaluate the impact of the MROT intervention on patient level outcomes including subsequent overdose and linkage to substance use disorder treatment. The R21 phase was completed in May 2019, and the R33 phase is being conducted from May 2019 to April 2022. This paper describes the protocol for the R33 outcomes portion of the study.

## Methods/design

### STR/SOR-funded MROT intervention model

The MROT intervention model was designed by the STR/SOR stakeholders and the MROT staff members, and the intervention is independent from the funded evaluation research. However, the STR/SOR program development, funding requirements, and oversight were heavily informed by the feasibility and acceptability research findings from the R21 phase of the MERIT study, and the research team and STR/SOR stakeholders met regularly to collaborate regarding optimizing the implementation of the intervention [4]. As part of the STR/SOR-funded intervention, participating EDs are provided with the phone number for the MROTs, which is triaged through a call-taking application that forwards the call to the mobile phones of all MROT team members’ individual phones. Upon identifying a potential patient, ED staff call the MROT team. The MROT team member who takes the call logs the nature of the call for internal documentation and drives to the ED to meet the patient. All MROT team members have undergone background checks and training in the participating hospitals and carry hospital credentials that allow them to enter the ED and meet with patients.

Upon arriving at the ED, the MROT team member speaks with the patient at bedside and offers a brief intervention as outlined in a standardized manual developed in the R21 phase by the MROTs and substance use disorder treatment experts. If the patient agrees, the MROT team member conducts a brief negotiated interview (BNI) designed to elicit the primary concerns of the patient, identify areas of need, and elicit motivation for change, loosely following the principles of Motivational Interviewing (MI). Based on the BNI, the team member provides a menu of services to the individual, including overdose education and naloxone distribution, linkage to care, referral to MAT, and community resources. If the patient refuses participation, the intervention does not take place.

For the purposes of the MERIT study, the MROT intervention consists of the single ED visit, though subsequent patient contact in the form of additional referrals, recovery support, and linkage to services may be provided following that initial visit. Subsequent contacts with the MROT are documented and included in the outcome analysis.

### MERIT study overview

The overarching goal of the MERIT study is to evaluate the effectiveness of the MROT intervention. We will accomplish this goal through two aims:

1. Determine the effectiveness of the MROT intervention on (a) subsequent overdose, and (b) MAT uptake among patients presenting to a hospital ED for opioid overdose or primary/secondary diagnosis of opioid use disorder.
2. Evaluate the MROT implementation and fidelity through a comprehensive process evaluation, including patient satisfaction interviews and fidelity monitoring.

Supplemental study activities include: 1) conducting interviews and participant observation to assess feasibility and acceptability of the MROT intervention in two rural/frontier hospitals not participating in the outcomes trial, and 2) using an Implementation Science approach to examine barriers and facilitators of successful program implementation in the 6 intervention hospitals.

Hospital participation in the intervention was “opt-in” based on agreements between hospital administration and the MROT program (i.e, the research team has no role in condition assignment). Six hospitals that agreed to participate in the MROT intervention will be designated as intervention hospitals, distributed across northern and southern Nevada and including larger hospitals in metropolitan areas and smaller hospitals serving outlying rural areas. Two rural hospitals will participate in the supplemental study activities to evaluate feasibility and acceptability of an alternative MROT model for rural/frontier hospitals. The remaining 29 hospitals in the state will be considered control hospitals for the purposes of the outcomes evaluation.

All study activities have been approved by the University of Nevada, Reno Institutional Review Board in accordance with the requirements of the Code of Federal Regulations on the Protection of Human Subjects (45 CFR 46 and 21 CFR 50 and 56) on March 15, 2018 (protocol number: 1204754). Any amendments to study activities, protocols, and procedures will be submitted to the University of Nevada, Reno Institutional Review Board for formal approval, and the decision will be provided to the principal investigator in writing. We have also obtained a Certificate of Confidentiality from the US Department of Health and Human Services, which provides additional privacy protections for participants involved in human subject research on sensitive topics such as substance use.

### Aim 1—outcome analysis data collection

There are two primary outcomes of interest that will be observed in the 9 months following the patient’s initial contact with the MROT: MAT initiation and non-fatal overdose. Secondary outcomes of interest include MAT retention and fatal overdose. To examine these outcomes, we will obtain de-identified data from the Nevada Division of Public and Behavioral Health Office of Public Health Informatics and Epidemiology (OPHIE) via a HIPAA compliant data sharing agreement. OPHIE receives identifiable patient data from all Nevada hospitals, all state-funded substance use disorder treatment providers, and the state’s coroners/medical examiners, and will compile a data set that includes all ED visits for opioid overdose/poisoning or primary/secondary diagnosis of opioid use disorder linked to data on treatment admissions, duration of treatment participation, and mortality. The data set will include ED visits beginning on September 1, 2019 and outcomes data will extend through the end of the project (to allow for a 6-month lag in reporting after the 9-month observation period). MROT staff will keep a log of every call received from the participating EDs, using a unique identification code for each patient, which will be matched to the ED data set to generate an indicator of patients that received the MROT intervention. The data set will be de-identified and shared with the research team using a Safe File Transfer Protocol server.

### Aim 1 –outcomes analysis plan

We hypothesize that in the 9 months following their ED visit, patients exposed to the MROT will:

H1: be more likely to initiate MAT (Primary outcome)
H2: have fewer non-fatal overdoses (ODs) (Primary outcome)
H3: be retained in MAT longer (Secondary outcome)
H4: be less likely to die of a subsequent ODs (Secondary outcome)

We estimate the possible number of opioid-related poisonings for the 6 intervention hospitals to be 1232. The upper bound of the possible number of participants is represented by the total set of opioid-related ED encounters, which was 2635 in the six intervention hospitals in 2017, representing 37% (2635/7125) of encounters statewide. Throughout the R33, we will continually collect data for all applicable participants seen in Nevada’s ED regardless of the lower and upper limits. We anticipate 5% of control patients will access MAT compared to 33% of intervention patients. We anticipate that 7–12% of control patients will experience a non-fatal overdose in the 9 months following their index overdose, and that will be reduced by around 47% among intervention participants. According to unpublished data from the Single State Agency, we anticipate 10% of control participants will die of an overdose in the next year compared to 5% in intervention participants. The alpha significance level of the power analysis is set to 0.05. At least 82% power will be achieved to detect a small effect size of 0.2 under the anticipated conservative sample size (achieving the generally accepted threshold of 80% or greater). Under different assumptions of effect size, such as medium effect size of 0.5 and large effect size of 0.8, our analysis strategy will have at least 99% power. For reasonably anticipated sample size 3316 (1232 intervention and 2084 control), at least 99% power will be achieved to detect as small as 0.1 effect size. Therefore, a very conservative sample of 829 (308 intervention and 521 control) patients will be sufficient to evaluate the primary outcomes. To be our best knowledge, very few existing power analysis approaches can be applied to hierarchical models. In addition to the primary aims of the proposal, data from this R33 portion will be used to develop a statistically reliable and computationally efficient power analysis procedure for hierarchical models and implement the proposed approach in an R package (to be developed, for R 3.6. 2) and a SAS macro (to be developed for SAS Version 9.4 for Windows). Patients will be considered intervention patients if they received services for opioid overdose or primary/secondary diagnosis of opioid use disorder (OUD) in the participating hospitals during the observation period. Patients seen for the same conditions in one of the 29 non-participating hospitals will be considered controls. Demographic and descriptive statistics will be calculated at baseline and at 9-months following the initial ED visit. The baseline characteristics and main outcomes between the intervention and control patients will be compared using chi-square tests and two-sample *t*-tests. 95% Confidence Intervals will be reported and all hypothesis testing will be two-sided. Analyses will be performed using SAS Version 9.4 for Windows.

To test the hypotheses with the dichotomous outcome (initiation of MAT) and the ordinal outcome (incidence of non-fatal OD) we will use generalized linear mixed effects models (SAS PROC GLIMMIX) and Bayesian hierarchical models (SAS PROC MCMC) to account for the complexity of non-normal outcomes and intricate cluster effects due to the interdependence of patients within hospitals. Sensitivity analysis for Bayesian hierarchical models will also be performed. For the time to treatment, retention in treatment, and death due to subsequent OD outcomes, we will apply marginal Cox models (SAS PROC PHREG) to estimate the main effect of intervention exposure and use the robust sandwich covariance matrix estimation approach to account for the intracluster dependence. Individual demographic information including age, race, sex, drug involved in the overdose (heroin vs. prescription opioids), insurance type (e.g., Medicaid vs. private), and location of residence (urban vs. rural) will be included in the mixed effects models and marginal Cox models to control for potential confounding effects. We will obtain records from OPHIE for 9 months prior to the index overdose for all participants, to allow us to control for prior history of non-fatal overdose and MAT utilization. Other covariates at the hospital level will include location (e.g., northern Nevada vs. southern Nevada, rural vs. urban) and hospital type (e.g., regional, university, trauma center, etc.).

#### Aim 2 –process evaluation–patient satisfaction interviews and fidelity monitoring

*Patient satisfaction interview recruitment*. Patients will be recruited to complete a brief post-intervention satisfaction interview via IRB-approved flyers distributed by the MROT team members, containing a phone number to call if participants are interested in participating in the interview. MROT staff will also ask patients if they would like to sign a release of information to allow the MROT team member to give patient contact information directly to the research team. Eligibility will be assessed using a brief screening questionnaire conducted over the phone. Eligibility criteria include: being over 18 years of age, having been seen for an opioid-related visit (overdose or primary/secondary diagnosis of opioid use disorder) in an intervention hospital in the last 30 days, and having met with an MROT staff member during that visit.

Study staff are located in northern and southern Nevada. Eligibility screening and interview scheduling will occur in the southern Nevada office, although interviewers are available in both locations to conduct in-person interviews at a location of the patient’s choosing (i.e., a study field office, hospital conference room, or semi-private location agreed to by the patient). After determining eligibility, an interview time and location will be established in a central paper appointment scheduler that will be maintained in the southern Nevada study office to avoid breaches of confidentiality and/or confusion in scheduling. If the patient lives in northern Nevada, the local staff member will be notified of the appointment date and time via phone. One day prior to, or the day of, the appointment a call will be made to the participant to confirm the appointment.

*Patient satisfaction interview procedures*. All participants will provide written informed consent according to the IRB-approved protocol. After the informed consent procedure, data will be collected via in-person interviews utilizing Qualtrics® software on a laptop computer or tablet that has been certified by the Federal Information Processing Standard (FIPS) Publication 140–2 security standard. Because of the mixed methods nature of the interview, in which we collect both quantitative survey data and qualitative narrative, the interviews will be recorded in their entirety using the device’s internal microphone (with permission from the participant). Recorded interviews will be transcribed verbatim by study staff and subjected to Quality Assurance procedures to ensure accuracy. Participants will be compensated $50.00 cash for their time and effort.

*Patient satisfaction interview measures*. The collection and analysis of quantitative (QUAN) and qualitative (QUAL) data will occur simultaneously in the same interview instrument. The surveys will begin with QUAL items designed to elicit narrative descriptions of the event of interest (i.e., meeting a mobile team in the hospital and all subsequent interactions following that event), followed with more specific QUAN questions including enrollment in social services, medication assisted treatment choices, subsequent overdoses and/or witnessing overdoses, and demographic information. In addition, QUAN scales such as MAT stigma, satisfaction with the encounter, and social support are included. We order the surveys in this way (QUAL then QUAN) because in other research we have identified a tendency for participants to provide shorter and less illustrative responses to QUAL questions if they have been asked to answer closed-ended QUAN questions at the beginning of the interview. We have also observed that the QUAL questions facilitate rapport-building between interviewer and respondent, which may increase the validity of responses to the QUAN questions.

*Patient satisfaction interviews analysis*. QUAL and QUAN data from the patient satisfaction interviews will be analyzed simultaneously, compared or related to each other, and interpreted together. First, we will describe the distribution of the QUAN variables using measures of frequency, central tendency, and dispersion. We will examine the psychometric properties of the multi-item MAT stigma construct by calculating Cronbach’s alpha to assess its reliability. Second, transcripts from the patient satisfaction interviews will be analyzed using an iterative thematic approach. We will review individual transcripts and interviewer field notes, making memos that document initial impressions and begin to synthesize observations. Based on this initial read, a set of initial “open codes” will be developed to identify *a priori* any emergent themes and will be applied to the entire set of notes and transcripts using Atlas.ti software for organization of thematic content. The codes will be reviewed and condensed into a set of hierarchically-arranged topics, and data will be extracted from Atlas.ti according to those codes. A second review of the coded data will identify any additional sub-themes or codes that should be added. Throughout the process, memos will be updated to document emergent understandings of the connections between themes.

#### Aim 2 –process evaluation–process data & fidelity monitoring

*Process evaluation and fidelity monitoring data collection and measures*. Intervention process data will be acquired from OPHIE as part of our data sharing agreement and directly from the MROTs. All data will be de-identified and tabulated. Fidelity monitoring data will be collected for descriptive purpose using two methods: MROT members will fill out a self-report fidelity assessment in Qualtrics®, and a trained research assistant will conduct fidelity observations of 10% of the intervention sessions using a structured observation checklist. The self-report and fidelity monitoring checklist includes an itemized list of intervention components, assessment of quality and proportion delivered of the brief negotiated interview, ability to provide patient with requested resources (including take home naloxone and ability to take next steps for treatment), any plans for future communication with the peer, and any additional notes.

*Process evaluation and fidelity monitoring data analysis*. Process data will be tabulated over the 9-month intervention period, including: the number of opioid overdose patients presenting in participating EDs, the number of patients who have made contact with the MROT staff, the number of patients receiving overdose education and naloxone from MROT staff, the number of referrals made to treatment services and/or MAT, the duration of MAT participation if enrolled, and the number of subsequent fatal and nonfatal overdoses among MROT patients. Fidelity monitoring data will be described using frequencies and measures of central tendency and dispersion.

#### Supplemental study activities

In addition to the primary outcomes and process evaluation described above, the MERIT study will: 1) evaluate the feasibility and acceptability of an alternative MROT model for rural/frontier EDs that do not have the same access to resources (including peer recovery support specialists, methadone clinics, and buprenorphine prescribers) as their urban counterparts; and 2) use an Implementation Science approach to examine the barriers and facilitators of successful program implementation in the six intervention hospitals participating in the outcomes evaluation trial.

#### Theoretical approach for supplemental activities

We will use Diffusion of Innovation (DOI) theory to inform the feasibility and acceptability research among the rural/frontier hospitals. This theory was developed based on empirical work demonstrating that there is a consistent pattern of adoption of new ideas in a system or organization [5]. DOI describes five characteristics of an innovation that characterize its adoptability: relative advantage, complexity, compatibility, trialability, and observability [6]. Using this framework, we will examine how the MROT intervention is described and received by ED staff, MROT team staff, key stakeholders in the hospitals and state agencies, and patients, with a focus on perceived usefulness (relative advantage) and ease of use (complexity). These two characteristics are especially important as they will provide the context needed to refine the intervention for future dissemination. Finally, potential barriers to adoption of the intervention will be explored as perceived by those inside the hospital organizations, people who use opioids, and MROT staff.

To inform the Implementation Science aim, we will use the Consolidated Framework for Implementation Research (CFIR). CFIR includes five domains and 39 constructs that are salient for implementing a successful innovation [7]. We will focus on those domains and constructs that were identified as critical to adoption in the formative phase of this research, including intervention characteristics (relative advantage, adaptability, complexity), inner setting (culture, relative priority, readiness for implementation, and leadership engagement), and process (planning, engaging, champions, and execution) [4].

#### Data collection for supplemental activities

Feasibility and acceptability interviews will be conducted with ED providers and MROT staff. ED providers will be recruited through IRB-approved flyers that will be disseminated through employee newsletters, professional organizations, one-on-one contact, and postings in the hospital’s employee break rooms. We will attempt to diversify the sample in terms of sex/gender and professional roles. MROT staff will be recruited through one-on-one contact. Implementation science interviews will be conducted with stakeholders who have played a prominent role in establishing MROT teams. Stakeholders will be recruited through a non-descript email. For all supplemental activity interviews, the IRB has approved a waiver of documentation of consent because the only piece of identifying information linking a participant to the study would be a signed consent form. Study staff will review an IRB-approved Information Sheet with participants and ask them to explicitly provide their verbal consent before proceeding with study activities.

ED and MROT surveys will include QUAL questions that will ask the respondents to describe how the MROT integrates into existing ED operations and QUAN questions that assess relative advantage, complexity, and compatibility of the MROTs in their current organizational structure. We will also ask them to rate their level of awareness, enthusiasm, and support for the MROT program. Stakeholder interviews will include QUAL questions that help frame the broader community context that existed before and during the MROT team rollout and the role individual stakeholders played in influencing the rollout. In addition, QUAN questions will assess whether MROTs are perceived as an effective strategy for the state response to the opioid overdose crisis.

To further examine the structural, cultural, and organizational factors that may affect MROT implementation, we will conduct participant observation in hospital EDs. Hospitals may refuse to participate in the participant observation; refusal will not affect provision of mobile team services. Participant observation will identify structural and cultural factors that influence the flow of information within hospital EDs. Ethnographic annotations will be derived using undisguised participant observations that include unstructured interviews, notes based on observations and interactions, and documentation of observed surroundings such as sketches of the environment itself by research personnel and/or MROT staff. Subjects to be observed include visual communication within workspaces (for example notices, signage, data display screens, noticeboards, and white boards), one-on-one interpersonal verbal communication amongst staff and between staff and patients (for example speech, gesture, affect, and tone), and stimuli in workspaces that have the potential to affect reception of information (for example sound, foot traffic, brightness, and spatial arrangement).

These observations and visual communication artifacts will be documented in field notes that will be coded and analyzed using visual data methodologies. Specifically, discursive visual analysis methods will be used to examine the production, use, and rhetorical composition of observed spaces by observed subjects [8]. Analyses will focus on identifying a) how information is disseminated broadly by the ED staff regarding the mobile teams in both physical locations in the hospital and through person-to-person communication efforts; b) barriers to receiving information about mobile teams in EDs; and c) opportunities for improved communication of information about mobile teams.

## Discussion

This study will be conducted according to the institutional guidelines set forth to conduct ethical and scientifically-sound research, and to adequately protect human subjects. Study activities, protocols, and any amendments will be submitted to the University of Nevada, Reno Institutional Review Board for formal approval, and the decision will be provided to the principal investigator in writing. The research efforts were funded by Arnold Ventures.

Scientific manuscripts will be developed to disseminate findings from this research. Results may also be shared with the scientific and public health community through meetings and conferences, or public facing communication materials such as summaries, infographics, or research briefs. We will apply the Ethical Visualization for Impact method to inform the responsible dissemination of our research findings (including scientific publications and any public-facing communication materials) [9]. This method recognizes the inherently rhetorical nature of scientific communication, and the potential for harm that may be unintentionally engendered by some communication materials. Consequently, Ethical Visualization applies a ‘do no harm’ philosophy to production of scientific communication materials. It involves six stages: discovery, impact analysis, framing, data shaping, visual production, and publishing and measuring impact. These six stages mitigate harm by considering intended and unintended consequences, engaging audiences in development of materials, consciously crafting the visual argument within dissemination materials, and assessing intended impact and measuring the actual impact of the materials. Outcomes will also be used to inform practical application of future peer-driven interventions, peer work in emergency departments and hospitals, and top-down dissemination of federal dollars to addresses public health crises. In addition, the MERIT team will continue to share outcomes with community partners and professionals.

## Supporting information

S1 File Step-by-step protocol. Also available on protocols.io.
(PDF)

## List of abbreviations

BNI: Brief negotiated interview
CFIR: Consolidated Framework for Implementation Research
DOI: Diffusion of Innovation
Eds: Emergency departments
FIPS: Federal Information Processing Standard
MAT: Medication assisted treatment
MERIT: Mobile Emergency Recovery Intervention Trial
MI: Motivational Interviewing
MROTs: Mobile recovery outreach teams
ODs: Overdoses
OPHIE: Office of Public Health Informatics and Epidemiology
OUD: Opioid use disorder
QUAN: Quantitative
QUAL: Qualitative
SAMHSA: Substance Abuse and Mental Health Administration
SOR: State Opioid Response
STR: State Targeted Response
