# Supplementary material for: The mobile emergency recovery intervention trial (MERIT): Protocol for a 3-year mixed methods observational study of mobile recovery outreach teams in Nevada’s emergency departments
Source: PLoS One. 2021 Oct 28;16(10):e0258795. doi: 10.1371/journal.pone.0258795 (PMC8553041; doi:10.1371/journal.pone.0258795)
Supplement: S1 File — Also available on protocols.io. (PDF) [file pone.0258795.s001.pdf]

Strategic Targeted Response for Opioid Overdose Evaluation

smith167 [1]

[1]University of Nevada, Reno

dx.doi.org/10.17504/protocols.io.br95m986

smith167

ABSTRACT

The Substance Abuse and Mental Health Administration awarded State Targeted Response grants to support states' efforts to address the opioid epidemic. In Nevada, one component of this grant was mobile recovery outreach teams (MROTs) that utilized peer recovery support specialists to provide care for qualifying patients in emergency departments (EDs). The Mobile Emergency Recovery Intervention Trial (MERIT) is a mixed methods study to assess the feasibility/acceptability and effectiveness of the MROT intervention. Data will be derived from state-level data sets containing de-identified emergency department visits, substance use disorder treatment records, and mortality files; in-person mixed methods interviews; participant observation; and self-report process evaluation forms. Primary outcomes include Medication Assisted Treatment (MAT) initiation and non-fatal overdose; secondary outcomes include MAT retention and fatal overdose. Quantitative hypotheses will be tested using generalized linear mixed effects models, Bayesian hierarchical models, and marginal Cox models. Qualitative interview data will be analyzed using an inductive thematic analysis procedure.

DOI

dx.doi.org/10.17504/protocols.io.br95m986

PROTOCOL CITATION

smith167 . Strategic Targeted Response for Opioid Overdose Evaluation. **protocols.io** https://protocols.io/view/strategic-targeted-response-for-opioid-overdose-ev-br95m986

LICENSE

This is an open access protocol distributed under the terms of the Creative Commons Attribution License, which permits unrestricted use, distribution, and reproduction in any medium, provided the original author and source are credited

CREATED

Feb 09, 2021

LAST MODIFIED

Feb 09, 2021

PROTOCOL INTEGER ID

47133

Description

The over-arching goal of this research is to test the effectiveness of a Mobile Recovery

1 Outreach Team (MROT) intervention for opioid overdose and/or opioid use disorder patients treated in Nevada's Emergency Departments (EDs). MROTs will provide peer coaching and mentorship, referrals to medication-assisted treatment (MAT), and opioid overdose education and naloxone as part of the State's SAMHSA STR Program. We will accomplish the research goal through the following two aims: (1) Determine the effectiveness of the MROT on (a) subsequent overdose, and (b) MAT uptake among patients presenting to a hospital ED for opioid overdose, and (2) Evaluate the MROT implementation and fidelity through comprehensive process evaluation. In addition, the research will examine the feasibility and acceptability of a mobile team intervention model in rural Nevada. Because we know Nevada has a unique landscape with many cities located in rural/frontier areas where a traditional model may not be feasible, we will expand our feasibility/acceptability research and outcomes evaluation to include examining the impact of alternative models in hospitals in the rural/frontier regions of Nevada.

Hypotheses

2 We hypothesize that in the 9 months following their index overdose, patients exposed to the MROT will: H1: be more likely than control participants to initiate MAT H2: be retained in MAT longer than control participants H3: have fewer non-fatal ODs than control participants H4: be less likely to die of a subsequent OD than control participants All analyses will be conducted using the intent-to-treat principle. Demographic and descriptive statistics will be calculated at baseline and at 9-months following the index overdose. The baseline characteristics and main outcomes between the intervention and control groups will be compared using the chi-square test and the two-sample t-test. 95% Confidence Intervals will be reported, and all hypothesis testing will be two-sided.

Design Plan

3 Data is collected from study subjects that are not randomly assigned to a treatment. This includes surveys, “natural experiments,” and regression discontinuity designs.

We will employ a quasi-experimental design to test the effectiveness of the MROT. Given the logistical and political considerations of evaluating the real-world implementation and effectiveness of a SAMHSA-funded program, a randomized controlled trial (RCT) is infeasible. Because this study is explicitly linked to, but not in total control over, the rollout of the SAMHSA-funded intervention, the study design must accommodate the fact that the SSA will implement a highly complex set of activities and is dependent on uptake of the program by each hospital ED. This logistical constraint makes it impossible to randomize en bloc. Therefore, a quasi-experimental design that rigorously controls for sources of confounding and bias is preferable. We anticipate there will be six hospital EDs in the implementation phase at the beginning of the study. Patients from these six hospitals who preset with opioid overdose or a primary or secondary diagnosis of Opioid Use Disorder (OUD) and have contact with the MROT will be the intervention participants. Patients who present with these conditions at the hospitals that have not yet adopted the MROT program will serve as the control participants. We will analyze the data using a traditional approach that controls for sources of confounding and bias, while adjusting for clustering within hospitals. Also, we will analyze the data using a Propensity Score Matching approach to mitigate bias and support our ability to make causal inferences. All patient data will be obtained as de-identified secondary data from our state partners.

Sampling Plan

4 Single state agency databases – Patient data from 3 databases maintained by the Nevada Department of Public and Behavioral Health will be linked at the state level, de-identified, and sent to the research team for quantitative analysis. The databases include: statewide hospitalization data, statewide mortality data, and statewide medication assisted treatment participant data, In person interviews with patients conducted after exposure to mobile outreach teams will be conducted by research assistants to access team interaction,

demographics, and drug use behaviors and impact of the intervention on patients. In person interviews for feasibility and acceptability will be conducted in rural Nevada with emergency department staff and other applicable community members that may be utilized for mobile recovery activities.

4.1 Sample Size:
In 2017, the six participating hospitals reported a total of 308 opioid-related poisoning visits to the ED, representing 37% (308/829) of the total visits statewide. Those numbers are highly conservative, because they are restricted to a small set of ICD-10 billing codes (primary diagnosis of T40.0, T40.1, T40.2, T40.3, T40.4, and T40.6). Other research found that using only ICD-9/10 billing codes related to opioid poisoning results in low sensitivity (as low as 25%). Additionally, rates of opioid-related visits have increased significantly over the past several years in Nevada (+12% 2010-2015, and +22% 2014-2015). Therefore, we anticipate that the actual number of intervention participants will be significantly higher. Using the findings from the sensitivity analysis above, we estimate the possible number of opioid-related poisonings for the 6 intervention hospitals to be 1232. The upper bound of the possible number of participants is represented by the total set of opioid-related ED encounters, which was 2635 in the six intervention hospitals in 2017, representing 37% (2635/7125) of encounters statewide. We anticipate 5% of control patients will access MAT compared to 33% of intervention patients. We anticipate that 7-12% of control patients will experience a non-fatal overdose in the 9 months following their index overdose, and that will be reduced by around 47% among intervention participants. According to unpublished data from the SSA, we anticipate 10% of control participants will die of an overdose in the next year compared to 5% in intervention participants.

4.2 Sample Size Rationale:
The alpha significance level of the power analysis is set to 0.05. At least 82% power will be achieved to detect a small effect size of 0.2 under the anticipated conservative sample size. Under different assumptions of effect size, such as medium effect size of 0.5 and large effect size of 0.8, our analysis strategy will have at least 99% power. For reasonably anticipated sample size 3316 (1232 intervention and 2084 control), at least 99% power will be achieved to detect as small as 0.1 effect size. Therefore, a very conservative sample of 829 (308 intervention and 521 control) patients will be sufficient to evaluate the primary outcomes. R package pwr was used for power analysis. To be our best knowledge, very few existing power analysis approaches can be applied to hierarchical models. In addition to the primary aims of the proposal, data will be used to develop a statistically reliable and computationally efficient power analysis procedure for hierarchical models and implement the proposed approach in an R package and a SAS macro.

4.3 Stopping Rule:
Throughout the study, we will continually collect data for all applicable participants seen in Nevada’s ED regardless of the lower and upper bounds of our planned sample size. We will terminate after we have fulfilled 9 months of follow-up observation for the first enrolled participant.

Measured Variables

5 Intervention vs. Standard of Care: The exposure of interest is whether patients receive the MROT program or standard of care. Standard of care will be defined as receiving standard discharge instructions and a passive referral to local services. There are 2 primary outcomes

of interest that will be observed in the 9 months following the patient's initial contact with the MROT: MAT treatment initiation, and non-fatal overdose. Secondary outcomes of interest include MAT treatment retention and fatal overdose. All outcome variables will be measured using data that are available within the SSA databases and which will be provided to the research team in a linked, de-identified data set. Non-fatal overdose will be defined as experiencing an overdose that is treated by ED personnel in the six months subsequent to the initial contact with the MROT. In 2017 Nevada passed AB474, new opioid-related legislation that classified opioid overdose as a reportable condition. This law took effect July 1, 2017 and requires that ED clinicians make a name-based report to the public health authority when they treat an opioid overdose. This mandate should ensure that opioid overdoses are identified in the state surveillance databases. MAT treatment uptake will be measured in three ways. We will obtain records from the SSA that contain information about all patients enrolled in MAT through a state-funded or state-certified provider. First, to measure treatment uptake we will define a dichotomous variable representing whether patients enrolled in a MAT program. Second, we will define "time to treatment" as the time elapsed between the index overdose and enrollment in the MAT program. Third, we will follow MAT patients for 9 months and determine the duration of treatment participation for enrolled patients. Fatal overdose will be defined as a death of a patient that is determined to be opioid related (ICD-10 codes X42, X44, Y12, Y14, T40.0 – T40.6), as reported in the electronic death registry system. We will include all intentional, unintentional, and undetermined deaths in the initial analysis, and will conduct sub-analyses in which only unintentional and undetermined deaths are included.

Analysis Plan

6 We hypothesize that in the 9 months following their index overdose, patients exposed to the MROT will: H1: be more likely to initiate MAT (Primary outcome) H2: have fewer non-fatal ODs (Primary outcome) H3: be retained in MAT longer (Secondary outcome) H4: be less likely to die of a subsequent OD (Secondary outcome) All analyses will be conducted using the intent-to-treat principle. Demographic and descriptive statistics will be calculated at baseline and at 9-months following the index overdose. The baseline characteristics and main outcomes between the intervention and control groups will be compared using the chi-square test and the two-sample t-test. To test the hypotheses with the dichotomous outcome (initiation of MAT) and the ordinal outcome (incidence of non-fatal OD) we will use generalized linear mixed effects models (SAS PROC GLIMMIX) and Bayesian hierarchical models (SAS PROC MCMC) to account for the complexity of non-normal outcomes and intricate cluster effects due to the interdependence of patients within hospitals. The sensitivity analysis for Bayesian hierarchical models will also be performed. For the time to treatment, retention in treatment, and death due to subsequent OD outcomes, we will apply marginal Cox models (SAS PROC PHREG) to estimate the main effect of intervention exposure and use the robust sandwich covariance matrix estimation approach to account for the intracluster dependence. Individual demographic information including age, race, sex, drug of choice (heroin vs. prescription opioids), insurance type (e.g., Medicaid vs. private), and location of residence (urban vs. rural) will be included in the mixed effects models and marginal Cox models to control for potential confounding effects. We will obtain records from the SSA for 9 months prior to the index overdose for all participants, to allow us to control for prior history of non-fatal overdose and MAT utilization. Other covariates at the hospital level will include location (e.g., Northern Nevada vs. Southern Nevada, rural vs. urban) and hospital type (e.g., regional, university, trauma center, etc.). In addition to the statistical approach described above, we will also employ a propensity score (PS) matching approach as a secondary analysis. PS matching is particularly well-suited to "natural experiments" in which assignment to condition cannot be manipulated, such as the implementation of state programs. The approach involves calculating the probability of assignment to a treatment or exposure using logistic regression in which we enter observed characteristics that could influence assignment. In this case, known characteristics of patients such as age, sex, location of residence (rural vs. urban), previous history of overdose, or previous history of MAT enrollment. The PS reduces the large set of possible covariates that could influence selection into the intervention or control

condition into a single summary variable. The PS represents the a priori probability that patients will be in the intervention or control condition; patients with similar PS's are assumed to have the same distribution of covariates. After calculation of the PS, intervention and control patients are 'matched' based on the overlap in their PS's, the distribution of covariates in the two groups compared, and success of the matching evaluated. Finally, the outcomes analysis is conducted using regression approaches as described above using the matched set of patients.

6.1 Inference Criteria:

A p-value of <0.05 will be considered statistically significant. 95% Confidence Intervals will be reported and all hypothesis testing will be two-sided. All analyses will be performed using SAS Version 9.4 for Windows.

6.2 Exploratory Analysis:

We will test a series of interaction terms to determine whether the effect of the intervention varies by subgroups. For example, it is possible the intervention may have different effects for women than men, or for heroin users compared to prescription opioid users. We also anticipate the intervention may have different effects depending on whether the patients are in a rural or urban area. To examine these subgroup differences, we will construct interaction terms that are the product of demographic variables and intervention condition (e.g., sex x condition, drug of choice x condition) and will repeat our outcomes analyses while including the interaction terms. If interaction terms are found to be statistically significant, we will stratify the sample and examine the intervention effect separately by subgroup.
